# *Trametes polyzona* as a Source for Bioremediation and Industrial Applications: A Systematic Review

**DOI:** 10.3390/jof12010019

**Published:** 2025-12-26

**Authors:** Melanie Ashley Ochoa-Ocampo, Maria Belén Macas-Granizo, Nina Espinosa de los Monteros-Silva, Thomas Garzón, Anthony Jose Balcazar-Sinailin, Zulay Niño-Ruiz, Roldán Torres-Gutiérrez, José R. Almeida, Noroska G. S. Mogollón, Karel Diéguez-Santana

**Affiliations:** 1 Laboratorio de Productos Naturales, Universidad Regional Amazónica Ikiam, Km 7 Via Muyuna, Tena 150101, Napo, Ecuador; melanie.ochoa@ikiam.edu.ec; 2 Department of Biomedical Sciences for Health, University of Milan, 20133 Milano, Italy; maria.macas@unimi.it; 3 Laboratorio de Biología Molecular y Bioquímica, Universidad Regional Amazónica Ikiam, Km 7 Via Muyuna, Tena 150101, Napo, Ecuador; nina.espinosadelosmonteros@ikiam.edu.ec; 4 Laboratorio de Química I, Universidad Regional Amazónica Ikiam, Km 7 Via Muyuna, Tena 150101, Napo, Ecuador; thomas.garzon@ikiam.edu.ec; 5 Life of Sciences Faculty, Universidad Regional Amazónica Ikiam, Km 7 Via Muyuna, Tena 150101, Napo, Ecuador; anthony.balcazar@est.ikiam.edu.ec (A.J.B.-S.); roldan.torres@ikiam.edu.ec (R.T.-G.); 6 Biomass to Resources Group, Universidad Regional Amazónica Ikiam, Km 7 Via Muyuna, Tena 150101, Napo, Ecuador; zulay.nino@ikiam.edu.ec; 7 Biomolecules Discovery Group, Universidad Regional Amazónica Ikiam, Km 7 Via Muyuna, Tena 150101, Napo, Ecuador; rafael.dealmeida@ikiam.edu.ec; 8 School of Pharmacy, University of Reading, Reading RG6 6UB, UK

**Keywords:** bioactive compounds, ligninolytic enzymes, white rot fungi, environment, health resources, biological products, biodegradation

## Abstract

*Trametes polyzona* is a white-rot basidiomycete with increasing relevance in environmental biotechnology due to its ligninolytic enzymes, biodegradation capacity, and versatile metabolic responses to diverse substrates. To provide an integrated and updated understanding of its biotechnological potential, we conducted a systematic review following PRISMA guidelines. A total of 46 studies published between 1991 and 2024 were analyzed, covering enzymatic production profiles, degradation of xenobiotics, extraction of bioactive metabolites, and experimental conditions influencing performance. Across the literature, *T. polyzona* consistently exhibits high ligninolytic activity, including laccase specific activities reported up to 1637 U/mg, together with efficient transformation of dyes, pesticides, and phenolic pollutants, and promising antioxidant and antimicrobial properties. However, substantial methodological heterogeneity was identified, particularly in strain characterization, fermentation parameters, and analytical approaches used to quantify enzymatic and biodegradation outcomes. These inconsistencies limit cross-study comparability and hinder process standardization. This review integrates current evidence; highlights critical gaps, such as limited ecotoxicological assessment of degradation products and scarce multi-omics characterization; and identifies key opportunities for process optimization in submerged/solid-state fermentation, bioreactor scaling, and the valorization of fungal metabolites. Overall, *T. polyzona* remains an underutilized resource with distinct advantages for applied mycology, environmental remediation, and industrial biotechnology.

## 1. Introduction

Fungi represent one of the most diverse and ecologically significant biological groups in both terrestrial and aquatic ecosystems [1]. Their role in biogeochemical cycles, symbiosis with plants and animals, as well as organic matter decomposition makes them essential for maintaining natural balance [2,3]. Moreover, from a biotechnological perspective, fungi have been widely used in the production of antibiotics, industrial enzymes, bioactive compounds, and fermented foods, demonstrating their versatility and scientific relevance [4]. In particular, white-rot fungi (WRF) have attracted significant attention in recent years, either for their valuable enzyme systems capable of efficiently degrading lignocellulosic biomass [5], or their ability to break down a wide range of pollutants generally found in industrial wastewater [6].

Among the WRF in the family Polyporaceae are members of the genus *Trametes*, which have been the subject of numerous studies due to their ecological, biotechnological, and medicinal relevance. One of the most outstanding species is *Trametes polyzona*, recognized for its efficiency in producing ligninolytic enzymes for applications in the paper and textile industries and for degrading environmental pollutants [7,8]. Therefore, it is recognized as one of the most effective decomposers of wood, typically found in association with hardwood species. Additionally, this species been reported to produce bioactive compounds with pharmacological potential—including antioxidant, antimicrobial and anticancer properties—which has generated growing interest in the scientific community [9,10].

Within the genus *Trametes*, *T. versicolor* is widely regarded as the benchmark species for ligninolytic activity and biotechnological applications, particularly in temperate environments [11]. However, emerging evidence suggests that *Trametes polyzona*, a predominantly pantropical species, exhibits several traits that may confer advantages under specific operational conditions. These include higher thermotolerance, distinct ligninolytic enzyme ratios with frequent laccase dominance, and robust growth on lignocellulosic and agro-industrial residues under nutrient-limited or high-temperature conditions [12,13]. Such characteristics position *T. polyzona* not merely as an alternative to *T. versicolor* but as a potentially more suitable candidate for applications requiring elevated temperatures, laccase-driven oxidation, or treatment of complex effluents in tropical and subtropical settings.

Belonging to that, Adongbede et al. [14] the analysis of antioxidant and antibacterial activity of phenolic extracts of *T. polyzona* strains found that the extracts exhibited comparable activity to antibiotics such as ceftazidime and erythromycin, inhibiting the growth of bacteria such as *Klebsiella pneumoniae*, *Escherichia coli*, *Staphylococcus aureus*, and *Salmonella enterica* [14]. In addition, crude polysaccharides from *T. polyzona* have demonstrated notable bioactivity. Kurniawan et al. [15] reported that these polysaccharides exhibit significant antioxidant capacity against 2,2′-azino-bis(3-ethylbenzothiazoline-6-sulfonic acid) (ABTS) radicals and possess antiproliferative effects against MCF-7 breast cancer (BC) cells, with an IC_50_ value of 0.58 mg/mL. Importantly, these compounds showed a favorable selectivity index, maintaining over 80% viability in normal human kidney cells (293) even at 1.6 mg/mL, suggesting a promising safety profile for potential anticancer development. These findings suggest potential antiproliferative, antioxidant, and apoptosis-inducing effects against the tested BC cell lines.

In the field of biotechnological and industrial applications, *T. polyzona* stands out for its ability to produce ligninolytic enzymes, such as laccases (Lac), manganese peroxidases (MnP) and lignin peroxidases (LiP). These enzymes are key for textile effluent decolorization, paper biopulping, biopolymer synthesis, and degradation of persistent organic compounds [7,16,17]. Furthermore, this fungus produces hydrolytic enzymes, such as cellulases, xylanases, and hemicellulases, which are essential for the degradation of lignocellulosic residues [18,19]. Recent studies have shown that *T. polyzona* can produce customized enzymes in situ, achieving up to 90% sugar recovery efficiency. This makes it a promising alternative to commercial enzymes for ethanol production from biomass [20]. These findings underscore its potential in biorefinery processes and biotechnology industry.

The bioremediation potential of *T. polyzona* is well established, primarily due to its ligninolytic enzyme system, which enables the degradation of various environmental pollutants such as synthetic dyes, phenolic compounds, polycyclic aromatic hydrocarbons (PAHs), and endocrine disruptor compounds (EDCs) [21,22,23]. It has been successfully used in the decolorization of textile effluents [8,19,24,25], degradation of PAHs [22], and treatment of agricultural and industrial wastewater [26,27]. Moreover, *T. polyzona* has shown effectiveness in removing estrogenic compounds from wastewater [28]. These applications position *T. polyzona* as an ecological and efficient solution for soil and water decontamination associated with industrial activities.

Despite the growing interest in *T. polyzona* for its biotechnological, medicinal, and bioremediation potential, significant gaps in the literature can hinder a comprehensive understanding of its applications. The available information is scattered across disciplines, limiting a structured synthesis of scientific advances in key areas such as biological activity, medicinal potential, industrial applications, and bioremediation potential. Additionally, there is a notable lack of comprehensive systematic reviews that synthesize the current state of knowledge on this species. Therefore, this study proposes a systematic review following the PRISMA methodology to organize and critically evaluate current scientific knowledge, providing a solid basis for future research and practical applications.

However, despite the growing number of studies on ligninolytic activity, biodegradation performance, and applied mycology, there is still no comprehensive and critical synthesis that integrates these capacities for *T. polyzona*. This review addresses this gap by systematically analyzing the available evidence, identifying methodological limitations, and highlighting the biotechnological potential of this species across environmental and industrial applications.

## 2. Materials and Methods

This study follows a systematic review approach to investigate the research landscape related to *Trametes polyzona*.

### 2.1. Search Strategy

Bibliographic data collection was carried out using the Scopus and Web of Science databases on 28 January 2025. We combined controlled keywords and synonyms related to *T. polyzona* using Boolean operators: for Scopus, the core string was TITLE-ABS-KEY (“*Trametes polyzona*” OR “*Coriolus polyzonus*” OR “*Coriolopsis polyzona*”), and for Web of Science we used TS = (“*Trametes polyzona*” OR “*Coriolus polyzonus*” OR “*Coriolopsis polyzona*”). Filters were applied for language (English), document type (‘article’ and ‘review’), and time range (1991–2024) to ensure the relevance of the results. The exact search strings and database-specific syntax are provided in Table A1 (Appendix A). The datasets from both databases were exported in RIS format, merged in EndNote X9 software, and checked for duplicate records using automated and manual screening.

### 2.2. Eligibility Criteria

Studies were eligible if they characterized *T. polyzona* or evaluated its biological, environmental, or industrial applications. Publications were excluded if they focused on other fungal species, were duplicate records, or did not provide primary information relevant to *T. polyzona*.

### 2.3. Study Selection

Two reviewers independently screened titles, abstracts, and full texts using the PRISMA 2020 protocol [29]. All screening decisions and reasons for exclusion were documented. An evaluation matrix was applied to rank the studies according to their relevance and methodological clarity. A completed PRISMA 2020 checklist is provided as Supplementary Table S2. This review was not registered, and no formal protocol was prepared.

### 2.4. Data Extraction

Data extraction was performed independently by two reviewers using a standardized template. For each study, information was collected on bibliographic details, fungal strain or source, fermentation or culture conditions, analytical methods, enzymatic activities, biodegradation parameters, and reported bioactive properties. All extracted data were cross-checked, and discrepancies were resolved by discussion until consensus was reached. No automation tools were used, and no study authors were contacted for additional information.

## 3. Results and Discussion

### 3.1. Study Selection Results

**Figure 1:**
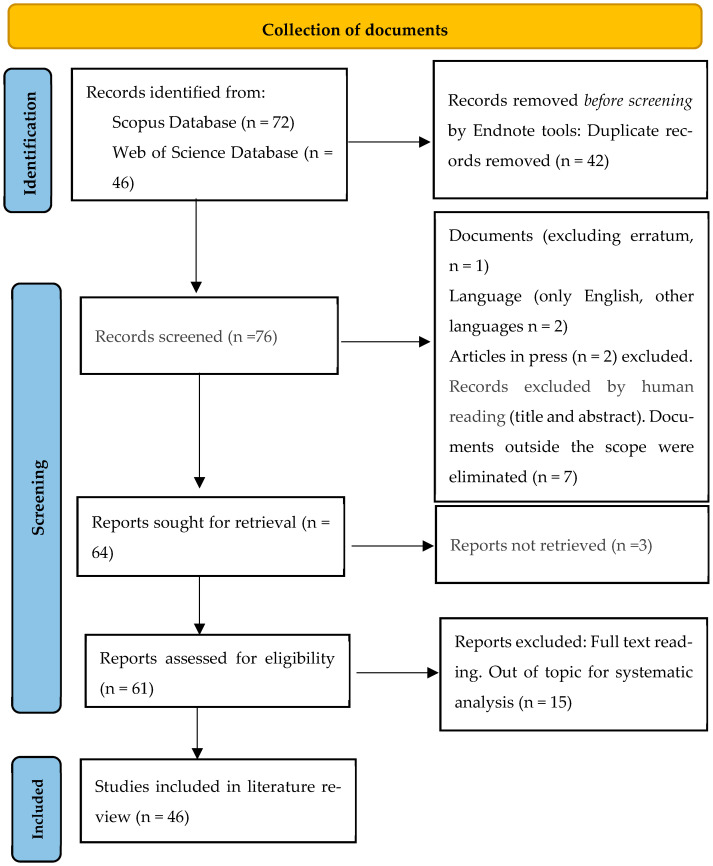
Flow diagram summarizing document selection procedure, adapted from PRISMA 2020 [29]. The completed PRISMA checklist is available as Supplementary Table S2.

The PRISMA 2020 flowchart (Figure 1) summarizes the results of the search across different databases and the number of documents selected at each stage. The eligibility criteria were defined before performing the search, and duplicate files found in WoS and Scopus (42 papers) were eliminated (leaving 76 documents). Subsequently, content analysis was performed by reading the complete article (abstract, methods, results, and conclusion). A total of five records were excluded due to erratum (*n* = 1), non-English languages (*n* = 2), or articles in press (*n* = 2). Subsequently, seven documents were eliminated because they were undoubtedly outside the scope of the review. In addition, three documents were removed due to the unavailability of the full text. After applying the inclusion and exclusion criteria, and following the screening process, a total of 46 relevant articles was retained for systematic analyses. A detailed list of excluded studies at the full-text stage, with reasons for their exclusion, is provided in Supplementary Table S1. A completed PRISMA 2020 checklist is provided as Supplementary Table S2.

### 3.2. Content Analysis. Systematic Literature Review

#### 3.2.1. General Characteristics

*Trametes polyzona* (Pers.) Justo (syn. *Coriolopsis polyzona*), originally described as *Polyporus polyzonus* [30], is a pantropical polypore characterized by a coriaceous appearance. It exhibits a reniform to flabelliform pileus, with a yellowish-ochre, tomentose to pilose, concentrically zoned surface. The pore surface is typically white to cream-colored.

This white-rot fungus efficiently degrades hardwood through the production ligninolytic enzymes (laccase, manganese peroxidase, lignin peroxidase) and cellulolytic enzymes, leading to spongy or fibrous decay [31,32]. *T. polyzona* has been widely studied for its biological activity and biotechnological potential, including its applications in the decolorization of olive oil mill wastewater [33], the degradation of synthetic dyes, and the removal of endocrine-disrupting chemicals.

Table 1 summarizes the compounds identified in extracts of *T. polyzona*, including their classification, molecular characteristics, relative abundances, and biological relevance.

The FAME profile is dominated by unsaturated fatty acids (84% relative abundance), including widespread components such as oleic, linoleic, and α-linolenic acids [9]. Although these fatty acids are not unique to *T. polyzona*, their relative proportions—especially the high unsaturated-to-saturated ratio—may contribute to the antioxidant and membrane-modulating activities observed in the extracts. More distinctive is the detection of heneicosanoic acid, a long-chain saturated fatty acid that is relatively rare in fungal lipids and could serve as a chemotaxonomic or functional marker in conjunction with other metabolite classes. In the aqueous extract, glucose is the predominant monosaccharide (97.34%), with minor arabinose and mannose contributions, consistent with a polysaccharide matrix that may underlie the reported immunomodulatory and antiproliferative effects [15].

A notable limitation in the literature on *T. polyzona* is the partial characterization of its chemical profile. Studies have focused mainly on primary metabolites, such as sugars and fatty acid esters (Table 1), which, although biologically relevant, do not fully explain the wide range of pharmacological activities attributed to the species. Therefore, it is suggested that future research prioritize the isolation and identification of secondary metabolites—terpenoids, alkaloids, and phenolic compounds—through bioactivity-guided fractionation and high-resolution mass spectrometry. This approach will allow the identification of the active principles responsible for antioxidant, antimicrobial, and anticancer activities, thus consolidating the therapeutic and biotechnological potential of the species.

#### 3.2.2. Biological Activities of *T. polyzona*

Table 2 presents a summary of the biological activity of extracts obtained from *T. polyzona*, including antioxidants, antibacterial, anticancer, antifungal and anti-inflammatory capabilities, and toxicity evaluations. Furthermore, Supplementary Table S3 outlines the key results associated with each activity.

The biological activities reported for *Trametes polyzona* have been linked to several classes of metabolites, although their chemical characterization remains partial in most studies. Analyses of lipophilic extracts by GC–MS have consistently shown a predominance of fatty acids, particularly unsaturated fatty acids such as oleic, linoleic, and α-linolenic acids, together with less common components such as heneicosanoic acid [9]. Although these fatty acids are not unique to *T. polyzona*, their relative abundance may contribute to the antibacterial and antioxidant activities observed, potentially through mechanisms involving membrane disruption and modulation of inflammatory processes.

In parallel, polar extracts—especially methanolic and phenolic-rich fractions—have demonstrated strong free-radical scavenging capacity in DPPH and ABTS assays [14,15]. In most cases, these extracts are characterized by total phenolic content rather than by the identification of individual phenolic compounds, suggesting that low-molecular-weight phenolics collectively account for the reported antioxidant activity. These same extracts have also exhibited notable antibacterial effects against both Gram-positive and Gram-negative bacteria, including *Staphylococcus aureus*, *Escherichia coli*, and *Salmonella enterica* [14,34], as well as antifungal activity against *Trichophyton mentagrophytes* and *Aspergillus fumigatus* [34]. The enhanced antimicrobial efficacy observed in extracts obtained under solid-state fermentation conditions further suggests that polar secondary metabolites play a central role in these bioactivities [9].

Beyond antioxidant and antimicrobial properties, *T. polyzona* has shown promising anticancer and anti-inflammatory potential. Water-soluble polysaccharide fractions isolated from mycelial cultures have demonstrated selective antiproliferative activity against MCF-7 breast cancer cells (IC_50_ = 0.58 mg/mL), while exhibiting low cytotoxicity toward normal cell lines [15]. These effects are commonly attributed to immunomodulatory and apoptosis-related mechanisms, although specific molecular targets have not yet been elucidated for *T. polyzona*. In addition, ethanolic extracts from fruiting bodies have displayed significant anti-inflammatory activity in animal models, achieving up to a 39.58% reduction in induced inflammation [10].

With respect to safety, available data indicate low acute toxicity of *T. polyzona* extracts in *Artemia salina* bioassays [10]. Nevertheless, despite these encouraging biological activities, the specific secondary metabolites responsible for each effect, their quantitative contribution, and their precise mechanisms of action remain insufficiently characterized. This underscores the need for comprehensive metabolomic profiling, bioactivity-guided fractionation, and mechanistic studies to accurately identify and validate the compounds underlying the antioxidant, antibacterial, anticancer, and anti-inflammatory properties attributed to *T. polyzona*.

#### 3.2.3. Enzymatic Activity of *T. polyzona*

Table 3 summarizes the enzymatic activities reported for *T. polyzona* strains under various fermentation conditions, substrates, and culture parameters. To complement this information, Supplementary Table S4 provides detailed activity data for each enzyme evaluated in the literature.

The studies highlight its ability to produce a wide range of hydrolytic and oxidative enzymes, such as cellulases (Cel), xylanases (Xyl), laccases (Lac), manganese peroxidases (MnP) and lignin peroxidases (LiP). These enzymes are useful in bioremediation, biofuel production, and other industrial applications.

Most studies characterize these enzymes at the biochemical level, reporting activity profiles, substrate specificity, optimal pH and temperature ranges, and molecular weights determined by SDS–PAGE. In contrast, molecular-level identification remains limited. To date, only one study has addressed gene-level evidence for ligninolytic enzymes in *T. polyzona*. Ezike et al. (2020) [35] screened the genome of *T. polyzona* WRF03 using laccase gene–specific primers targeting conserved copper-binding regions, obtaining a positive PCR amplification of an approximately 1500–1600 bp fragment, which is consistent with the expected size of fungal laccase genes. This result confirms the presence of a laccase-encoding gene in *T. polyzona*.

However, the amplified sequence was not fully characterized, and no gene sequences were deposited in public databases. At present, complete gene sequences for laccase, manganese peroxidase, or lignin peroxidase from *T. polyzona* are not available. Consequently, current knowledge of the enzymatic system of this species relies largely on biochemical characterization, and the lack of genomic and transcriptomic data represents a significant gap that limits comparative analyses and enzyme engineering efforts.

**Table 3:** Enzymatic activity of *T. polyzona* on different substrates and fermentation conditions.

Fungus/Strain	Substrate	Fermentation Type ^a^	Culture Conditions ^b^	Enzyme Activity	Reported Yield/Titer (Maximum)	Method of Analysis ^c^	Ref.
*C. polyzona* MUCL 38443	Tree leaves	SSF	pH = 6, T = 27 °C, t = 7–14 days	CMC, Xyl, FPA, Lac, MnP	CMC: 9 U/mL	DNS; ABTS; Phenol red (UV–Vis)	[36]
*C. polyzona* MUCL 38443	Wheat straw	SSF	pH = 6, T = 27 °C, t = 7–14 days	CMC, Xyl, FPA, Lac, MnP	CMC: 5 U/mL	DNS; ABTS; Phenol red (UV–Vis)	[36]
*C. polyzona* MUCL 38443	Apple peels	SSF	pH = 6, T = 27 °C, t = 7–14 days	CMC, Xyl, FPA, Lac, MnP	CMC: 16 U/mL	DNS; ABTS; Phenol red (UV–Vis)	[36]
*C. polyzona* MUCL 38443	Banana peels	SSF	pH = 6, T = 27 °C, t = 7–14 days	CMC, Xyl, FPA, Lac, MnP	CMC: 21 U/mL	DNS; ABTS; Phenol red (UV–Vis)	[36]
*C. polyzona* MUCL 38443	Tree leaves	SmF	pH = 6, T = 27 °C, t = 3–10 days	CMC, Xyl, FPA, Lac, MnP	Lac: 1.78 U/mL	DNS; ABTS; Phenol red (UV–Vis)	[36]
*C. polyzona* MUCL 38443	Mandarin peels	SmF	pH = 6, T = 27 °C, t = 3–10 days	CMC, Xyl, FPA, Lac, MnP	CMC: 94 U/mL	DNS; ABTS; Phenol red (UV–Vis)	[36]
*T. polyzona* BKW-001	Cassava peels	SSF	pH = 5, T = 30 °C, t = 14 days	EG, β-Glu, Exg, Xyl, Amy, Cel	Amy: 56.2 U/mL	HPLC/PAHBAH (UV–Vis)	[20]
*T. polyzona* BKW-001	Cassava peels	SSF	pH = 6, T = 30 °C	EG, β-Glu, Exg, Xyl, Amy	EG: 1.97 U/mL	PAHBAH (UV–Vis)	[37]
*T. polyzona* BKW-001	Cocoa pod husk	SSF	pH = 6, T = 30 °C	EG, β-Glu, Exg, Xyl, Amy	Amy: 0.9 U/mL	PAHBAH (UV–Vis)	[37]
*T. polyzona* BKW-001	Water Hyacinth	SSF	pH = 6, T = 30 °C	EG, β-Glu, Exg, Xyl, Amy	Amy: 0.4 U/mL	PAHBAH (UV–Vis)	[37]
*T. polyzona* HHM001	Corn leaf residues	SSF	T = 37 °C, t = 11–15 days	Lac, LiP, MnP, Cel, Xyl	Lac: 80 U/mL	ABTS; Veratryl alcohol; Phenol red	[19]
*C. polyzona* CCBAS 740	Wheat straw	SSF	pH = 4.5, T = 28 °C, t = 25 days	Lac, MnP	MnP: 22 U/mL	MBTH + DMAB (UV–Vis)	[38]
*T. polyzona* WRF03	ABTS	SSF	pH = 4.5, T = 55 °C, t = 9 days	Lac (purified)	Lac: 1637 U/mg protein (66 kDa)	SDS-PAGE; ABTS	[39]
*T. polyzona* KU-RNW027	ABTS/guaiacol	NR	pH = 4.5, T = 50 °C	MnP, Lac (purified)	MnP: 16.5 U/mg protein	SDS-PAGE; UV–Vis	[17]
*T. polyzona* WR710–1	Tangerine orange peels	SSF	pH = 2.2, T = 50 °C	Lac	Lac: 1.36 U/mg/71 kDa (SDS-PAGE)/68 kDa (Gel filtration)	Oxidation of 2,6-DMP/SDS–PAGE/Gel filtration	[37]
*T. polyzona*	Fruit nopal paddle	SSF	T = 28 °C, t = 5 days	Cel, Xyl	Xyl: 0.0036 U/mg	DNS (UV–Vis)	[15]
*T. polyzona* MPS1-3	Palm oil mill effluent	NR	pH = 4, T = 37 °C, t = 10 days	Lac	Lac: 156.3 U/mL	SDS-PAGE	[40]
*T. polyzona* MUCL 38443	ABTS	SSF	pH = 6, T = 50 °C, t = 20 days	Lac	Lac: 0.97 U/mL. MM: ~60 kDa	SDS-PAGE; ABTS	[13]
*T. polyzona*	Melanin	NR	pH = 5.3, T = 25 °C	Lac	Lac: 2.42 U/mL	UV–Vis	[41]
*T. polyzona* RYNF13	Wood meal guaiacol agar	SmF	pH = 6, T = 30 °C, t = 16 days	MnP	MnP: 24 U/mL	Oxidation of 2,6-DMP	[21]
*C. polyzona* CCBAS 740	NR	NR	T = 28 °C, t = 7 days	Lac, LiP, MnP	MnP = 0.056 U/mL	SDS-PAGE; ABTS	[42]
*T. polyzona* MUCL 38443	ABTS	NR	pH = 3, T = 70 °C	Lac	Lac: 800 U/L	SDS-PAGE; ABTS	[43]
*C. polyzona* MUCL38443	Olive Oil Mill Wastewater	SmF	pH = 5, T = 30 °C, t = 21 days	Lac, LiP, MnP	Lac = 0.038 U/mL	UV–Vis	[44]
*T. polyzona*	Effuent with Amaranth dye	SmF	t = 10 days	Lac, LiP, MnP	LiP: 0.038 U/mL	ABTS; Veratryl alcohol; Phenol red	[25]
co-culture of *T. polyzona*, *A. niger*, *T. longibrachiatum*, *M. circinelloides* and *R. microsporus*	Pharmaceuticals	SBR	pH = 3–4.6, t = 6 days	Lac, LiP, MnP	MnP: 0.253 U/mL	ABTS; 2,6-DMP	[28]

Notes ^a^: Type of Fermentation. SSF: Solid-state fermentation, SmF: Submerged fermentation. NR: Not reported. SBR: Sequencing batch reactor. ^b^ Culture Conditions. T: Temperature, t: time. ^c^ Method of Analysis. ABTS: 2,2′-azino-bis(3-ethylbenzothiazoline-6-sulfonic acid), DNS: 3,5-dinitrosalicylic acid, PAHBAH: para-hydroxybenzoic acid hydrazide, MBTH: 3-methyl-2-benzothiazolinone hydrazone hydrochloride, DMAB: 3-dimethylaminobenzoic acid, 2,6-DMP: 2,6-dimethoxyphenol, SDS-PAGE: Sodium dodecyl sulfate-polyacrylamide gel electrophoresis. HPLC: High Performance Liquid Chromatography.

Fermentation and Substrate Influence: As shown in Table 3, SSF and SmF affect enzyme yield differently. SmF generally yields higher enzyme production than SSF, particularly for ligninolytic enzymes like Lac and MnP, due to better nutrient diffusion and oxygen availability. For example, SmF on tree leaves resulted in Lac and MnP activities of 1780 U/L and 896 U/L, respectively, significantly higher than the levels obtained via SSF (Table S4) [36]. The substrate composition critically affects enzyme yield. Fruit residues like mandarin peels induce high cellulase activity (94 U/mL), while banana peels are effective for laccase production [36]. Cassava peels, rich in starch, induce high amylase activity (56.22 U/mL) (Table S4) [20].

Optimization Parameters: Culture conditions such as pH, temperature, and aeration significantly influence enzyme production. Optimal laccase production of 156.3 U/mL was achieved at pH 4 and 37 °C [40]. Enhanced aeration and reduced substrate particle size also increase enzyme yield [19].

Enzyme Characterization: Enzyme molecular masses were determined using methods like SDS-PAGE, revealing sizes such as 66 kDa for Lac [39] and 42–44 kDa for MnP [17]. Analytical techniques for activity measurement include UV-Vis spectrophotometry with substrate-specific assays like ABTS oxidation for laccase and the DNS method for cellulases [36].

In summary, the enzymatic activity of *T. polyzona* is highly dependent on fermentation type, substrate selection, and culture conditions. SmF and lignocellulosic substrates like fruit residues and tree leaves generally enhance enzyme production, making this fungus a promising candidate for industrial bioprocesses [36].

#### 3.2.4. Molecular Mechanisms and Critical Comparison Between Studies

The degradative ability of *Trametes polyzona* and other white-rot fungi relies on the synergistic action of three major enzyme groups: laccases (Lac), manganese peroxidases (MnP), and lignin peroxidases (LiP) [45,46]. Laccases catalyze one-electron oxidations of phenolic substrates, generating phenoxyl radicals that trigger β–O–4 bond cleavage or lignin repolymerization. Their oxidative range toward non-phenolic compounds is substantially expanded by laccase–mediator systems that use mediators such as ABTS or HBT to shuttle electrons between the enzyme and otherwise inaccessible substrates [47].

MnPs oxidize Mn^2+^ to Mn^3+^, which forms Mn^3+^–organic acid complexes (e.g., oxalate or malonate) that diffuse into the extracellular matrix and oxidize phenolic compounds at a distance from the catalytic site [48]. LiPs, possessing the highest redox potential (~1.2 V), oxidize non-phenolic lignin units and highly recalcitrant pollutants such as bisphenols and azo dyes [49]. These electrochemical and substrate differences explain the temporal expression patterns reported in several studies, where LiP predominates in early growth phases and Lac/MnP dominate during later stages [50].

For *T. polyzona* specifically, mechanistic insights are still fragmentary and are often extrapolated from studies on *T. versicolor* and other *Trametes* species. Nevertheless, several strain-level features have been reported. *T. polyzona* KU-RNW027 produces two MnP isoenzymes (42–44 kDa) and one laccase (71 kDa) with high redox potential and activity optima at pH 4.5 and 50 °C [17], while strain WRF03 secretes a 66 kDa laccase that remains active at 55 °C [39]. These molecular-weight patterns and temperature optima suggest a set of moderately thermotolerant ligninolytic isoenzymes that may partly explain the strong performance of *T. polyzona* in dye and pharmaceutical degradation (Table 3). However, without gene-sequence data or structural information, most mechanistic models remain inferred from better-studied *Trametes* species.

In *T. polyzona*, distinct isoenzymatic profiles are strongly correlated with degradation performance. Lueangjaroenkit et al. [17] characterized two MnPs and one laccase with unique catalytic properties, achieving >80% decolorization of azo dyes within 48–72 h. Chairin et al. [16] demonstrated that adding redox mediators significantly enhanced removal of non-phenolic dyes by *T. polyzona*. Together, these findings show that the Lac/MnP/LiP ratio is a practical predictor of oxidative capacity and may explain the wide range of efficiencies (40–95%) reported across studies depending on the target compound, substrate composition, pH, and nutrient balance [7,51].

Another major source of variability arises from methodological differences. Conventional spectrophotometric assays using model substrates (ABTS, guaiacol) often underestimate total enzymatic potential or overlook intermediate metabolites that can be identified only by chromatographic techniques (LC–MS or GC–MS). Therefore, a combined standardization of enzyme-activity assays with LC–MS/MS metabolomic profiling is essential to produce comparable datasets and to verify detoxification rather than simple decolorization.

Overall, this comparative synthesis highlights that *T. polyzona’s* oxidative efficiency results from a dynamic balance among Lac, MnP, and LiP activities, modulated by environmental parameters and oxidative stress during fermentation, consistent with mechanistic models previously proposed for *T. versicolor* and related *Trametes* species [51,52], while emphasizing the need for *T. polyzona*-specific omics and structural studies.

#### 3.2.5. Biodegradation Potential of *T. polyzona*

Table 4 shows the results of studies on the pollutant degradation capacity of *T. polyzona*, including synthetic dyes, pharmaceuticals, PAHs, and industrial effluents.

*Trametes polyzona* demonstrates a significant capacity to degrade a wide spectrum of industrial pollutants, including synthetic dyes, pharmaceuticals, polycyclic aromatic hydrocarbons (PAHs), and endocrine-disrupting compounds (EDCs), primarily through the action of its lignin-modifying enzymes (Lac, MnP, LiP) [17,21,28].

Degradation of Diverse Pollutants: The fungus is highly effective against various synthetic dyes, particularly anthraquinone dyes like Remazol Brilliant Blue R, which can be completely decolorized within 30 min [17]. The degradation of azo dyes is generally less efficient but can be enhanced using redox mediators like acetosyringone (AS) or 1-hydroxybenzotriazole (HBT) [13,16].

An important caveat in dye-removal studies is the relative contribution of biosorption versus enzymatic degradation. Only a minority of the reviewed works performed desorption tests or mass-balance analyses to differentiate dye molecules irreversibly transformed by enzymes from those merely adsorbed onto fungal biomass. Most studies report color loss based on UV–Vis absorbance without systematically quantifying desorbed dyes after washing the mycelium, meaning that the extent of true biodegradation may be overestimated in some cases (e.g., [56,57]). This methodological limitation applies to several of the dye-removal studies summarized in Table 4. Future experiments with *T. polyzona* should therefore incorporate standardized desorption controls, TOC/COD balances, and LC–MS/MS identification of intermediates to clearly disentangle biosorption from enzymatic transformation.

For pharmaceutical contaminants, *T. polyzona* can achieve complete removal of tetracyclines and certain non-steroidal anti-inflammatory drugs like ibuprofen [12,17]. The efficiency is notably higher in co-culture systems, where removal rates for compounds like carbamazepine can reach over 97% [28].

The fungus also degrades PAHs, with one strain showing 100% degradation of phenanthrene and 86% of pyrene [21]. Furthermore, it efficiently removes EDCs, achieving complete degradation of bisphenol A (BPA) and nonylphenol, especially when laccase is immobilized to enhance stability [16,55].

Application in Industrial Effluent Treatment: *T. polyzona* has been applied to treat complex industrial wastewaters, such as palm oil and olive oil mill effluents, achieving substantial reductions in chemical oxygen demand (COD), color, and phenolic compounds [26,40,44].

**Figure 2:**
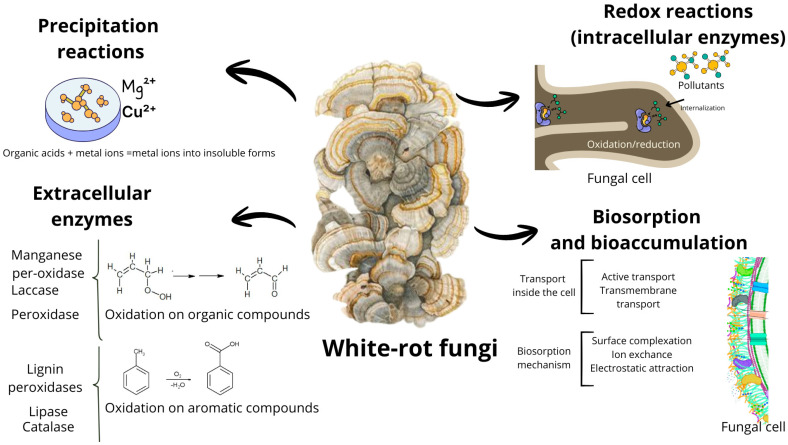
Mechanisms of fungal bioremediation (Adapted from [57,58]). Notes. Schematic representation of the bioremediation mechanisms of white rot fungi. The arrows indicate the direction of biochemical and transport processes, while the colored dots represent organic contaminants and metals. Precipitation reactions, extracellular and intracellular oxidation, as well as biosorption and bioaccumulation processes at the cell wall and fungal interior levels are shown.

Although the specific degradation mechanisms of different contaminant classes by *T. polyzona* have not yet been fully elucidated, available experimental evidence combined with established models for white-rot fungi allows the main transformation pathways to be summarized (Figure 2) [57]. For endocrine-disrupting compounds (EDCs) such as bisphenol A (BPA) and nonylphenol, laccase-mediated oxidation appears to be the primary initial step. This process involves the generation of phenoxy radicals, which undergo radical coupling reactions (dimerization and oligomerization), followed by side-chain hydroxylation and partial aromatic ring cleavage, as evidenced by LC–MS and GC–MS analyses [16,54,55]. While these transformations often reduce estrogenic activity, they may also lead to the formation of partially oxidized aromatic by-products with residual toxicity.

In the case of polycyclic aromatic hydrocarbons (PAHs), such as phenanthrene and pyrene, degradation is mainly driven by manganese peroxidase (MnP) and lignin peroxidase (LiP) through one-electron oxidation reactions that yield epoxides and quinone-type intermediates. These compounds can subsequently undergo ring-cleavage reactions, forming smaller, more polar metabolites such as carboxylic acids that are more amenable to further biodegradation or mineralization [21,23].

Emerging contaminants including pharmaceuticals—such as antibiotics (tetracyclines, quinolones) and non-steroidal anti-inflammatory drugs (diclofenac, ibuprofen)—are typically attacked at phenolic, aromatic, or amino functional groups, resulting in hydroxylated, dechlorinated, or otherwise oxidized derivatives [12,17,28]. In several cases, degradation efficiency is enhanced by redox mediators or co-culture systems, suggesting that the relative activities of Lac, MnP, and LiP strongly influence the distribution of transformation products.

It should be noted that many of these pathways are partly inferred from mechanistic studies on related white-rot fungi, such as *Trametes versicolor*. Nevertheless, the available data indicate that *T. polyzona* follows comparable oxidative strategies, with the Lac/MnP/LiP ratio, mediator availability, and culture conditions modulating degradation efficiency and intermediate formation. Importantly, several studies have reported residual toxicity of degradation products toward organisms such as *Daphnia magna*, *Vibrio fischeri*, and *Artemia*, highlighting that high contaminant removal does not necessarily equate to complete detoxification. Therefore, coupling chemical analyses with ecotoxicological assays is essential to accurately assess the environmental safety of *T. polyzona*-based bioremediation processes.

It should be noted that the efficiency of enzymatic degradation by *T. polyzona* is strongly influenced by operational parameters, particularly pH and temperature. Most studies report optimal ligninolytic activity under acidic conditions (pH 4–6) and moderate temperatures (25–30 °C), although some strains exhibit partial thermotolerance [21,35]. Variability in reported degradation efficiencies across studies can therefore be attributed not only to differences in contaminant structure, but also to strain-specific genetic variation, substrate complexity, culture conditions, and analytical methodologies.

### 3.3. Applications of T. polyzona

As mentioned above, the properties of *T. polyzona* have been extensively researched, with many applications ranging from biotechnology, industrial, and environmental science to nanotechnology and other emerging areas.

Environmental Bioremediation and Wastewater Treatment: One of the most prominent applications of *T. polyzona* is in the bioremediation of polluted environments. The fungus’s efficient ligninolytic system, particularly laccase (Lac), manganese peroxidase (MnP), and lignin peroxidase (LiP), allows for the degradation of a wide spectrum of recalcitrant pollutants. As evidenced in Table 4, this includes the successful decolorization of synthetic dyes from textile effluents (e.g., Remazol Brilliant Blue R and Amaranth dye [8,16,17,24,25,42], the breakdown of endocrine-disrupting chemicals (EDCs) like bisphenol A (BPA) and nonylphenol [16,54,55], and the removal of pharmaceuticals and personal care products, including antibiotics and anti-inflammatories [12,17,28]). Furthermore, *T. polyzona* demonstrates a robust capacity to degrade polycyclic aromatic hydrocarbons (PAHs) such as phenanthrene and fluoranthene [21,23], aligning it as a powerful tool for rehabilitating soils and waters contaminated by industrial waste and fossil fuels.

Bioenergy and Biorefining: In the context of the circular bioeconomy, *T. polyzona* offers substantial value in the valorization of lignocellulosic biomass. The fungus produces a suite of hydrolytic enzymes, including cellulases, xylanases, and amylases (Table 3), which efficiently break down agricultural residues like cassava peels, banana peels, and other agro-industrial waste [20,36,37]. This enzymatic hydrolysis facilitates the release of fermentable sugars for conversion into bioethanol, as demonstrated by Acheampong et al. [37]. This application not only provides a renewable energy source but also promotes the efficient and sustainable use of low-value agricultural by-products.

Pharmaceutical and Biomedical Potential: Beyond environmental applications, *T. polyzona* exhibits considerable promise in the health sector. Extracts from its fruiting bodies and mycelia have demonstrated a range of bioactivities (Table 2). These include significant antioxidant properties [14,15], potent antimicrobial effects against pathogens like *Staphylococcus aureus* and *Escherichia coli* [9,14,34], and notable antifungal activity against species such as *Trichophyton mentagrophytes* [34]. Moreover, studies have confirmed anti-inflammatory effects in animal models [10] and antiproliferative activity against cancer cell lines, such as MCF-7 breast cancer cells [15]. The bioactive compounds underlying these effects, which include unsaturated fatty acids and polysaccharides (Table 1), present a promising foundation for the development of nutraceuticals, natural antibiotics, and adjuvant therapies.

When compared with the extensively studied *T. versicolor*, *T. polyzona* exhibits several potentially advantageous traits for applied biotechnology. Multiple studies report sustained growth and enzymatic activity at temperatures between 35 and 40 °C, exceeding the typical optimum reported for *T. versicolor*. In addition, *T. polyzona* frequently displays a high laccase-to-peroxidase ratio, which can be advantageous in peroxide-independent oxidation processes such as dye decolorization, phenolic compound removal, and green synthesis applications. Rapid colonization of hardwood substrates and agricultural residues, together with tolerance to metal ions and complex effluents, further supports its suitability for bioremediation in warm climates and high-strength waste streams. While strain-level variability remains significant, these features suggest that *T. polyzona* can complement or outperform other *Trametes* species under specific operational constraints.

### 3.4. Process Limitations, Scalability, and Enzymatic Stability

The industrial viability of *T. polyzona*–based bioprocesses depends on balancing enzyme production cost, catalytic efficiency, and operational robustness. In SSF using low-cost lignocellulosic residues such as bagasse, sawdust, or rice straw, *T. polyzona* can yield 19.72 U/mg of laccase and up to 16.53 U/mg of MnP with minimal energy input [17]. In contrast, SmF generally produces lower yields (0.080–0.19 U/mL of laccase) but provides better control of aeration and pH, enabling reproducibility and integration into continuous-flow bioreactors [36,59].

The formulation and recovery of fungal enzymes may account for up to 50% of total operational costs in bioremediation [60,61]. Immobilization using natural polymers such as alginate, chitosan, or biochar, and the production of cross-linked enzyme aggregates (CLEAs) [62], can extend enzymatic lifetime for 10–12 reuse cycles [7], reducing treatment cost per unit. Nevertheless, support regeneration and partial catalytic loss (20–40%) remain significant barriers for industrial scaling.

At the reactor scale, *T. polyzona* shows tolerance to moderate fluctuations in pH and pollutant load. In airlift and packed-bed systems, decolorization efficiencies exceeding 80% for dyes and phenolic pollutants have been achieved after seven days of continuous operation [24,25]. However, scaling up is still limited by (i) oxygen transfer control needed to maintain Lac/MnP balance, (ii) foam formation and mycelial aggregation that restrict mass transfer, and (iii) progressive enzyme inactivation caused by radical accumulation.

Recent work indicates that biofilm-based reactors or porous carriers (e.g., coconut fibers, expanded silica) can improve enzyme stability and enable semi-continuous operation [24,63]. Co-immobilization with natural mediators such as sinapic acid or acetosyringone can further enhance electron-transfer efficiency while lowering dependence on synthetic mediators.

Despite its strong bioremediation potential, several challenges limit the large-scale application of *T. polyzona*. These include the structural recalcitrance of certain pollutants (e.g., methylene blue), the possible formation of toxic degradation intermediates, and the need to reduce enzyme production costs while optimizing reactor design for industrial implementation [25,35,55]. Addressing these challenges through enzyme immobilization, process optimization, and integrated toxicity assessment will be critical for translating laboratory-scale efficiencies into robust and economically viable bioremediation systems.

Ligninolytic enzymes are highly sensitive to extreme pH (<4.0 or >8.0), heavy-metal ions, and excess H_2_O_2_. *Trametes* laccases may lose up to 60% of their activity after ten reuse cycles under continuous flow [64]. Protein-engineering approaches—particularly directed evolution—have produced thermostable laccase variants in other fungal species [60], although recombinant *T. polyzona* enzymes remain unreported.

Incomplete degradation may yield intermediate products more toxic than the parent pollutants. For instance, triclosan and azo-dye degradation can generate chlorinated aromatic amines or oligomers with higher acute toxicity [65]. In *T. polyzona*, partially oxidized aromatic metabolites have been detected post-treatment, with residual toxicity confirmed by *Daphnia magna* and *Vibrio fischeri* assays [23,25]. This aspect is often overlooked in conventional chemical oxidation processes (e.g., Fenton, ozone), which can rapidly decolorize effluents but frequently increase toxicity by generating reactive electrophiles. In contrast, fungal treatments offer the possibility of coupling enzymatic oxidation with subsequent mineralization, provided that intermediate metabolites are rigorously monitored. Therefore, industrial scaling must integrate LC–MS/MS metabolite profiling and standardized ecotoxicological assays (e.g., Microtox^®^, *Daphnia magna*) to demonstrate true detoxification rather than mere color removal before wastewater discharge [66].

Collectively, these insights show that while *T. polyzona* presents clear advantages for eco-efficient bioprocesses, successful implementation depends on the simultaneous optimization of enzyme production, reactor design, and environmental safety validation.

### 3.5. Emerging and Cross-Sectoral Applications

The technological potential of *T. polyzona* continues to expand through advances in bioprocessing and nanobiotechnology. Enzyme-immobilization strategies, such as the formation of CLEAs have enhanced laccase stability and enabled its operation in continuous reactors for the removal of EDC and industrial dyes [54,55]. Moreover, the covalent attachment of *T. polyzona* laccase to silica or magnetic nanoparticles has shown efficient degradation of BPA in aqueous matrices, demonstrating potential for advanced nanocomposite-based wastewater treatment [67].

The broad application spectrum of *T. polyzona* reflects its versatile enzymatic system and metabolic adaptability. However, translating these promising laboratory-scale results into standardized industrial processes requires addressing persistent challenges, including strain variability, culture-mode differences (SSF vs. SmF), and extraction methodologies. Future efforts should prioritize protocol standardization, detailed metabolite profiling, and pilot-scale validation of immobilized enzyme systems. By systematically overcoming these constraints, *T. polyzona* can be fully harnessed as a sustainable and multifunctional biocatalyst, reinforcing its role in green chemistry, environmental protection, and biotechnological innovation.

### 3.6. Critical Appraisal of the Evidence

Despite the broad range of studies available, the evidence on *T. polyzona* remains highly heterogeneous and presents several methodological weaknesses that limit cross-study comparability. Most publications differ substantially in strain identification, culture conditions, and substrate preprocessing (e.g., use of raw versus steam-exploded biomass, chemical delignification, or size reduction below 5 mm), which hinders the establishment of reliable benchmarks for enzymatic activity or biodegradation performance. In many cases, experimental designs lack standardized controls, quantitative kinetic parameters, or adequate reporting of environmental variables such as pH, aeration, or nutrient availability. Furthermore, only a minority of studies identify intermediate metabolites or assess the post-treatment ecotoxicity of degradation products—an essential step for validating the environmental safety of fungal bioprocesses. Analytical methods also vary widely, from basic spectrophotometric measurements to more advanced LC-MS/MS approaches, creating inconsistencies in the detection and quantification of by-products. These gaps highlight the need for harmonized methodologies, improved analytical resolution, and more robust experimental designs to enable meaningful comparisons and support the advancement of *T. polyzona* as a reliable biotechnological platform. For example, studies employing finely milled, pre-treated agricultural residues often report higher apparent enzymatic activities and degradation efficiencies than those using coarse, untreated substrates, making direct comparison difficult in the absence of standardized preprocessing protocols.

## 4. Conclusions

This systematic review provides an integrated and critical synthesis of the current knowledge on *Trametes polyzona*, highlighting its strong potential for environmental and industrial biotechnology. The species consistently demonstrates high ligninolytic activity, effective transformation of structurally diverse xenobiotics, and the capacity to produce metabolites with antioxidant and antimicrobial properties. Collectively, the available evidence confirms that *T. polyzona* is a versatile fungal resource capable of contributing to bioremediation, enzymatic bioprocessing, and value-added applications.

However, the analysis also reveals substantial limitations in the existing body of research. Most studies rely on heterogeneous experimental conditions, insufficient strain characterization, inconsistent reporting of fermentation parameters, and analytical methods that prevent direct comparison of enzymatic or biodegradation efficiencies. Additionally, few studies evaluate ecotoxicity of degradation products or address the stability, kinetics, and scalability of fungal processes. These methodological gaps underline the need for more standardized protocols and robust analytical frameworks.

Future research should prioritize: (i) detailed characterization of strains and enzymatic isoforms; (ii) standardized experimental designs in submerged and solid-state fermentation; (iii) comprehensive monitoring of intermediate metabolites and post-treatment toxicity; (iv) integration of omics tools to elucidate regulatory pathways; and (v) pilot-scale studies that validate process performance under realistic operational conditions. Strengthening these areas will enable more reliable assessments of the organism’s biotechnological potential and facilitate translation from laboratory findings to industrial applications.

Overall, *T. polyzona* should be regarded as an underexploited but strategically relevant member of the genus, offering advantages over the reference species *T. versicolor* in thermotolerant, laccase-centered, and high-load bioremediation applications. Targeted comparative studies and multi-omics analyses will be essential to fully define its niche within industrial biotechnology.

## Figures and Tables

**Table 1:** Bioactive compounds present in extracts obtained from *T. polyzona.* Data derived from Fatty Acid Methyl Esters (FAMEs) identified by GC-MS in ethanolic extracts [9], and Monosaccharides identified by HPLC in aqueous extracts [12].

No.	Class	Compound Name	Molecular Formula	MW (g/mol)	Area (%)	Biological Activity
Fatty Acid Methyl Esters (FAMEs) * (ethanolic extract)						
1	Saturated (C8:0)	Caprylic acid methyl ester	C_9_H_18_O_2_	158.24	0.7430	Antimicrobial, precursor for flavor compounds
2	Saturated (C13:0)	Tridecanoic acid methyl ester	C_14_H_28_O_2_	228.37	2.3991	Rare in nature
3	Monounsaturated (C14:1Δ9)	Myristoleic acid methyl ester	C_15_H_28_O_2_	240.38	1.2098	Potential anti-inflammatory effects
4	Monounsaturated (C15:1Δ10)	*cis*-10-Pentadecanoic acid methyl ester	C_16_H_30_O_2_	254.41	2.5627	Found in dairy fats; role in membrane fluidity
5	Monounsaturated (C16:1Δ9)	Palmitoleic acid methyl ester (9-Hexadecenoic acid)	C_17_H_32_O_2_	268.43	1.3090	Omega-7; improves insulin sensitivity, anti-inflammatory
6	Saturated (C17:0)	Heptadecanoic acid methyl ester	C_18_H_36_O_2_	284.48	4.0211	Biomarker for dairy intake; minor metabolic roles
7	Saturated (C18:0)	Stearic acid methyl ester	C_19_H_38_O_2_	298.50	2.6906	Neutral effect on cholesterol; used in food emulsifiers
8	Monounsaturated *trans* (C18:1Δ9t)	Elaidic acid methyl ester	C_19_H_36_O_2_	296.49	5.6737	Trans fat; linked to cardiovascular disease
9	Monounsaturated *cis* (C18:1Δ9c)	Oleic acid methyl ester	C_19_H_36_O_2_	296.49	2.7858	Omega-9; heart-healthy, reduces LDL cholesterol
10	Polyunsaturated *trans* (C18:2Δ9t,12t)	Linolelaidic acid methyl ester	C_19_H_34_O_2_	294.47	12.4574	Trans fat; adverse metabolic effects
11	Polyunsaturated (C18:3Δ6,9,12)	γ-Linolenic acid methyl ester	C_19_H_32_O_2_	292.46	2.7134	Omega-6; anti-inflammatory, used in eczema treatment
12	Polyunsaturated (C18:3Δ9,12,15)	α-Linolenic acid methyl ester	C_19_H_32_O_2_	292.46	1.4846	Omega-3; essential fatty acid; supports brain health
13	Saturated (C21:0)	Heneicosanoic acid methyl ester	C_22_H_44_O_2_	340.58	5.8626	Rare; used in lipidomics research
14	Polyunsaturated (C20:2Δ11,14)	*cis*-11,14-Eicosadienoic acid methyl ester	C_22_H_38_O_2_	322.52	4.3872	Precursor for signaling molecules
Monosaccharide ** (aqueous extract)						
15	Hexose (C6)	Glucose	C_6_H_12_O_6_	180.16	97.34	Primary energy source; central in metabolism
16	Pentose (C5)	Arabinose	C_5_H_10_O_5_	150.13	2.52	Component of plant cell walls; used in food additives
17	Hexose (C6)	Mannose	C_6_H_12_O_6_	180.16	0.15	Immune modulation; precursor for glycoproteins

Notes. * Area (%) expressed in relative area and determined by GC-MS analysis [9]. ** Area (%) expressed only in monosaccharide composition and determined by high-performance liquid chromatography [15].

**Table 2:** Biological activity of the extracts obtained from the *T. polyzona*.

Biological Activity	Part/Strain	Process ^a^	Solvent Used	Extraction (Time/Temperature)	Assay Measure/Organism-Cell Line ^b^	Refs.
Antioxidant capacity	Fruiting bodies/*T. polyzona*	Raw	Acidified methanol	T = 23 °C, (magnetic stirrer at 2.5 Hz), t = 1 h	TPC, TEAC, DPPH	[14]
	Mycelial/*T. polyzona* CU07	Fermentation (T = 25 °C, t = 14 days, static)	Water	Reflux refractor (solid-to-liquid ratio of 1:40 (g/mL), T = 90 °C and t = 4 h)	DPPH, ABTS	[15]
	Fruiting bodies/*C. polyzona*	Raw	Methanol	T = 25 °C, t = 8 h	DPPH	[34]
Antibacterial	Fruiting bodies/*C. polyzona*	Raw	Dichlormethane/Methanol/Water	T= 25 °C, t = 8 h	IZ/*Staphylococcus aureus* (ATCC 29213), *Bacillus subtilis* (ATCC 6059), *Escherichia coli* (ATCC 25922), *Pseudomonas aeruginosa* (ATCC 27853)*,* and *Micrococcus flavus* (SBUG 16)	[34]
Antibacterial	Fruiting bodies/*T. polyzona*	Raw	Acidified methanol	T = 23 °C	IZ/*Klebsiella pneumoniae* (ATCC 1100975, ATCC 1100975, BAA1705, and ATCC 1100770). *E. coli*. (ATCC 700972, ATCC 25922, ATCC 25927). *Salmonella enterica*, *S. aureus* (ATCC 700698)	[14]
Antibacterial	Fruiting bodies/*T. polyzona*	Raw (UFM) and fermented (SSF, SmF). Fermentation: 4 days	Acetone and methanol	t = 3 days (occasional stirring)	MIC/*S. aureus* isolated from blood	[9]
Anticancer	Mycelial/*T. polyzona* CU07	Fermentation (T = 25 °C, t = 14 days, static)	Water	Reflux refractor (solid-to-liquid ratio of 1:40 (g/mL), T = 90 °C, t = 4 h)	Cell line MCF-7 BC (ATCC HTB-22)	[15]
Antifungal	Fruiting bodies/*C. polyzona*	Raw	Dichloromethane, methanol, and water	T = 25 °C, t = 8 h	IZ/*Candida maltosa* SBUG17, *Candida albicans* ATCC 90028, *Candida krusei* ATCC 90878, *Aspergillus fumigatus* 13550/99, *Mucor* sp., *Microsporum gypseum*, *Trichophyton mentagrophytes* 05/2004	[34]
Anti-inflammatory	Fruiting bodies/*C. polyzona*	Raw	Ethanol	T = 25 °C, t = 2 days	TPA-induced ear edema in mice	[10]
Lipid peroxidation	Fruiting bodies/*C. polyzona*	Raw	Ethanol	T = 25 °C, t = 2 days	TBARS levels were measured using rat brain homogenates	[10]
Toxicity	Fruiting bodies/*C. polyzona*	Raw	Ethanol	T = 25 °C, t = 2 days	Acute toxicity test/*Artemia salina*	[10]

Notes. ^a^ Process. SSF: Solid state fermentation, SmF: Submerged fermentation, UFM: Not fermented. ^b^ Assay Measure/Organism-Cell Line. TPC: Total phenolic content. TEAC: Trolox Equivalent Antioxidant Capacity. DPPH: 1,1-diphenyl-1-picrylhydrazil or 2,2-diphenyl-1-picrylhydrazil inhibition assay. ABTS: 2,2′-azino-bis(3-ethylbenzothiazoline-6-sulfonic acid) inhibition assay. MIC: Minimum inhibitory concentration. IZ: Inhibition zones. TPA: 12-O-tetradecanoyphorbol-13-acetate. TBARS: Thiobarbituric acid reactive species.

**Table 4:** Enzymatic biodegradation of industrial pollutants by *T. polyzona*.

Fungus/Enzyme ^a^	Contaminant/Colorant/Class ^b^	Initial Concentration	Degradation Conditions ^c^	Degradation Time	Eff. (%) ^d^	Method of Analysis ^e^	Refs.
*T. polyzona* KU-RNW027/MnP-Lac	Remazol Brilliant Blue R (RBBR)/Anthraquinone	25 mg/L	pH = 5, T = 30 °C, Agitation = 100 rpm. Enzyme = 1 U/mL	30 min	100	Spectrophotometry UV-Vis	[17]
	Reactive Blue 120 (RNB)/Azo dye			7 days	83–100		
	Reactive Yellow 160 (RBY)/Azo dye				69–73		
	Reactive Orange 107 (RGY)/Azo dye				33–46		
	Reactive Red 198 (RR)/Azo dye				42–75		
	Reactive Red 180 (RBR)/Azo dye				33–46		
*T. polyzona* WRF03/Lac	Coomassie Brilliant Blue (CBB)/Triphenylmethane	200 mg/mL	pH = 4.5, T = 25 °C. 0.5 mL of purified enzyme solution	6 h	72.35	Spectrophotometry UV-Vis	[35]
	Malachite Green/Triarylmethane				57.84		
	Methyl Orange (MO)/Azo dye				47.55		
	Erichrome Black (EB)/Azo dye				40.20		
	Congo Red (CR)/Diazo dye				18.11		
	Azure B (AB)/Thiazin				1.78		
	Methylene Blue (MB)/Heterocyclic				0.38		
*T. polyzona* KU-RNW027/MnP-Lac	Tetracycline/Tetracyclines	25 mg/L	pH = 4.5, T = 30 °C. Enzyme = 1 U/mL	1–3 days	100	LC-MS/MS	[17]
	Doxycycline/Tetracyclines			1–3 days	100		
	Amoxicillin/β-lactams			5 days	25–100		
	Ciprofloxacin/Quinolones			7 days	6.7–73		
*T. polyzona* WR710-1/Lac	Bisphenol A (BPA)/Benzene and substituted derivatives	0.01%	pH = 4, T = 28 °C, dark conditions. Enzyme = 0.64 U/mL	3 h	100	GC-MS + HPLC	[16]
	Bromophenol Blue (BRB)/Triphenlymethane		pH = 4, T = 28 °C, dark conditions. Enzyme = 0.45 U/mL + Redox mediator (HBT = 2 mM)	1 day	100	Spectrophotometry UV-Vis	
	Remazol Brilliant Blue R (RBBR)/Antraquinone				~96–98		
	Methyl Orange (MO)/Azo dye		pH = 4, T = 28 °C, dark conditions.		~88–95		
	Relative Black 5 (RB5)/Diazo dye		pH = 4, T = 28 °C, dark conditions. Enzyme = 0.45 U/mL + Redox mediator (HBT = 2 mM)		~50–90		
	Congo Red (CR)/Diazo dye				~70–85		
	Acridine Orange (AO)/Heterocyclic				~30–55		
*T. polyzona* MPS1-3 r	Palm Oil Mill Eluent/COD	61,100 mg/L	pH = 4.03, T = 28 °C, Agitation = 120 rpm	5 days	16.03	Spectrophotometry UV-Vis	[40]
	Palm Oil Mill Eluent/TSS	27,550 mg/L			70.15	Gravimetry	
	Palm Oil Mill Eluent/TS	45, 300 mg/L			38.9		
	Palm Oil Mill Eluent/Total phenolics compound	129.80 mg/L			50.84	Spectrophotometry UV-Vis + HPLC	
*T. polyzona* MUCL 38443/Lac	Amido Black (AB) 10B/Azo dye	50 mg/L	T = 50 °C, Agitation = 160 rpm. Enzyme = 0.11 U/mL + mediator (AS = 0.05 mM)	1 day	94.6	Spectrophotometry UV-Vis	[13]
	Bromocresol Purple Sodium Salt/Triphenlymethane			5 h	72.2		
	Orange G (OG)/Azo dye				79.3		
	Malachite Green Oxalate/Triarylmethane				94.6		
*T. polyzona* RYNF13/MnP, Lac, LiP	Phenanthrene/PAH	100 mg/L	pH = 6, T = 30 °C	11 days	100	Spectrophotometry UV Vis	[21]
	Fluorene/PAH				100		
	Pyrene/PAH				86		
*T. polyzona* LMB-TM5	Levafix Yellow E-3RL (LY-3RL)/Azo dye	400 mg/L	pH = 6, T = 28 °C	2 days	<20	Spectrophotometry UV Vis	[24]
	Remazol Brilliant Red 3BS (RBR-3BS)/Azo dye				<20		
	Remazol Brilliant Blue R (RBBR)/Anthraquinone				97		
	Cibacron Deep Red S-B (CDR-SB)/Azo dye			3 days	33.7		
	Synozol Yellow HF-4GL (SY-HF4GL)/Azo dye			1 day	<20		
	Synozol Turquoise Blue HF-G (STB-HFG)/Phthalocyanine				80		
	Real textile effluent			2 days	93	HPLC	
*T. polyzona* PBURU 12/Lac	Phenanthrene/PAH	100 ppm	T = 25 °C, dark conditions, Agitation = 150 rpm, Crude enzyme = 10 U/mL.	1 day	98	Spectrophotometry UV Vis/GC-MS	[23]
			T = 25 °C, dark conditions, Agitation = 150 rpm. Live culture (Submerged).	11 days	88		
*T. polyzona*/Lac, LiP, MnP	Amaranth dye (AM)/Azo dye	100–200 mg/L	Submerged 3 L reactor, T = 28 °C, Agitation = 150 rpm	21–27 days	100	Spectrophotometry UV Vis	[8]
	Orange G (OG)/Azo dye			27 days	92–93.5		
	Denim blue (commercial dye)			16–17 days	99		
*T. polyzona* isolated H18	Remazol Brilliant Blue R (RBBR)/Anthraquinone	100 mg/L	pH = 4,5, T = 25–30 °C	4 days	95.4	Oxidation of ABTS/Spectrophotometry UV Vis	[53]
	Acid Blue 129 (AB129)/Anthraquinone				89		
	Acid Orange 7 (AO7)/Monoazo dye				77.7		
	Reactive Black 5 (RB5)/Diazo dye				94.8		
*C. polyzona* MUCL 38443/Lac	Nonylphenol/EDC	5 mg/L	pH = 5, T = 50 °C. Enzyme = 1 U/L	8 h	100	HPLC-MS-ESI	[54]
	Bisphenol A/EDC				100		
	Triclosan/PCDE				65		
*C. polyzona* CCBAS 740/LiP, MnP, MnIP, Lac	Delor 106/PCB commercial mixture	0.9 ppm	T = 28 °C	21 days	41	GC-ECD	[42]
	Poly R-478/Triarylmethane	200 mg/L		3 days	100	Spectrophotometry UV Vis	
	Remazol Brilliant Blue R (RBBR)/Anthraquinone			10 days			
*C. polyzona* MUCL38443/LiP, MnP, Lac	Olive Oil Mill Wastewater/COD	25,000–100,000 mg/L	pH = 5, T = 30 °C, Static cultures/Agitation = 150 rpm.	21 days	31.3–59.1/50.1	Spectrophotometry UV Vis	[44]
	Olive Oil Mill Wastewater/Color			15 days	57.3–75.2/39.4		
*C. polyzona* MUCL 38443	Olive Oil Mill Wastewater/COD	102 g/L	pH 5.2, T = 25 °C	24 days	77	Spectrophotometry UV-Vis + HPLC	[26]
	Olive Oil Mill Wastewater/Phenols	3500 mg/L			91		
	Olive Oil Mill Wastewater/Color	-			63		
*T. polyzona.*	Amaranth dye (AM) (100 mg/L)/COD	1600 mg/L	Reactor Airlift, T = 37 °C	30 days	95.5	Spectrophotometry UV Vis	[25]
	Amaranth dye (AM)/Azo dye	100 mg/L		25 days	95		
*C. polyzona* MUCL 38443/Lac	Nonylphenol/EDC	5 mg/L	pH = 5, T = 20 °C. Reactor packed with Lac = 0.37 U/g	100 min	100	GC-MS	[55]
	Bisphenol A/EDC		pH = 5, T = 20 °C. Reactor packed with Lac = 0.75 U/g	200 min			
	Triclosan/PCDE			120 min			
co-culture of *T. polyzona*, *A. niger*, *T. longibrachiatum*, *M. circinelloides* and *R. microsporus*/Lac, LiP, MnP	Carbamazepine/Pharmaceutical compounds	1 mg/L	pH = 6.22, T = 25 °C. SBR at steady state, Agitation = 120 rpm	2 days	97.41	SPE-UPLC-QToF/MS	[28]
	Diclofenac/Pharmaceutical compounds				99.83		
	Ibuprofen/Pharmaceutical compounds				99.91		
*T. polyzona*/Lac, LiP, MnP	Carbamazepine/Pharmaceutical compounds	1 mg/L	pH = 4.3, T = 37 ± 1.5 °C	3 days	22	SPE-UPLC/MS	[12]
	Diclofenac/Pharmaceutical compounds			1 day	92		
	Ibuprofen/Pharmaceutical compounds			5 days	100		

Notes ^a^: Fungus/Enzyme. Lac: Laccase, MnP: Manganese Peroxidase, LiP: Lignin Peroxidase. ^b^ Contaminant/Colorant/Class. PAH: Polycyclic Aromatic Hydrocarbons, PCDE: Polychlorinated Diphenyl Ethers, COD: Chemical oxygen demand. PCBs: Polychlorinated biphenyls. ^c^ Degradation Conditions. AS: Acetosyringone, HBT: 1-hydroxybenzotriazole, SBR: Sequencing batch reactor. ^d^ Eff: Efficiency of Degradation/decolorization (%). ^e^ Method of Analysis. ABTS: 2,2′-azino-bis(3-ethylbenzothiazoline-6-sulfonic acid), HPLC: High Performance Liquid Chromatography. SPE-UPLC-QToF/MS: Solid-Phase Extraction-Ultra Performance Liquid Chromatography-Quadrupole Time-of-Flight Mass Spectrometry, HPLC-MS-ESI: High Performance Liquid Chromatography-Mass Spectrometry with Electrospray Ionization, GC-ECD: Gas Chromatography-Electron Capture Detection, SPE-UPLC/MS: Solid-Phase Extraction-Ultra Performance Liquid Chromatography/Mass Spectrometry, LC-MS/MS: Liquid Chromatography-Tandem Mass Spectrometry, GC-MS: Gas Chromatography-Mass Spectrometry.

## Data Availability

No new data were created or analyzed in this study. Data sharing is not applicable to this article.

## Supplementary Materials

The following supporting information can be downloaded at https://www.mdpi.com/article/10.3390/jof12010019/s1, Table S1: Studies excluded during the screening process and reasons for exclusion; Table S2: PRISMA 2020 Checklist: Locations of reporting items within the manuscript. Table S3: Summary of the main findings on the biological activity of *T. polyzona* extracts reported in the reviewed studies. Table S4: Enzymatic activity data by enzyme for each evaluated *T. polyzona* strain.

## Abbreviations

The following abbreviations are used in this manuscript:

ABTS	2,2′-azino-bis(3-ethylbenzothiazoline-6-sulfonic acid)
AM	Amaranth dye
Amy	Amylase
AS	Acetosyringone
BC	Breast Cancer
β-Glu	Beta-Glucosidase
Cel	Cellulase
CLEAs	Cross-linked enzyme aggregates
CMC	Carboxymethyl cellulase
COD	Chemical Oxygen Demand
DPPH	2,2-diphenyl-1-picrylhydrazyl
EDCs	Endocrine Disrupting Chemicals
EG	Endoglucanase
Exg	Exoglucanase
FAME	Fatty Acid Methyl Esters
FPA	Filter Paper Activity
GC-MS	Gas Chromatography-Mass Spectrometry
GC-ECD	Gas Chromatography-Electron Capture Detection
HBT	1-hydroxybenzotriazole
HPLC	High Performance Liquid Chromatography
HPLC-MS-ESI	High Performance Liquid Chromatography-Mass Spectrometry with Electrospray Ionization
IZ	Inhibition Zone
Lac	Laccase
LC-MS/MS	Liquid Chromatography-Tandem Mass Spectrometry
LiP	Lignin Peroxidase
MB	Methylene Blue
MC	Moisture content
MIC	Minimum Inhibitory Concentration
MM	Molecular Mass
MnP	Manganese Peroxidase
MnIP	Manganese Independent Peroxidase
PAHs	Polycyclic Aromatic Hydrocarbons
PCBs	Polychlorinated Biphenyls
PCDE	Polychlorinated Diphenyl Ethers
PS	Particle size
RBBR	Remazol Brilliant Blue R
SBR	Sequencing Batch Reactor
SDS-PAGE	Sodium Dodecyl Sulfate–Polyacrylamide Gel Electrophoresis
SmF	Submerged Fermentation
SPE-UPLC-QToF/MS	Solid-Phase Extraction-Ultra Performance Liquid Chromatography-Quadrupole Time-of-Flight Mass Spectrometry
SSF	Solid-State Fermentation
TBARS	Thiobarbituric Acid Reactive Substances
TEAC	Trolox Equivalent Antioxidant Capacity
TPC	Total Phenolic Content
TS	Total Solids
TSS	Total Suspended Solids
UFM	UnFermented Material
WoS	Web of Science
WRF	White Rot Fungi
Xyl	Xylanase

## Appendix A

**Table A1:** The specifics of search strings.

Database	Search Strings
SCOPUS	(TITLE-ABS-KEY (trametes AND polyzona) OR TITLE-ABS-KEY (polystictus AND polyzonus) OR TITLE-ABS-KEY (coriolus AND polyzonus) OR TITLE-ABS-KEY (polystictus AND septosporus) OR TITLE-ABS-KEY (coriolopsis AND polyzona)) AND (EXCLUDE (DOCTYPE, “er”)) AND (EXCLUDE (PUBSTAGE, “aip”)) AND (LIMIT-TO (LANGUAGE, “English”))
Web of Science (WoS)	(TS = (ALL= (Trametes polyzona)) OR ALL = (coriolopsis polyzona)) AND (LIMIT-TO (LANGUAGE, “English, Spanish”))
