# Supplementary material for: Trametes polyzona as a Source for Bioremediation and Industrial Applications: A Systematic Review
Source: J Fungi (Basel). 2025 Dec 26;12(1):19. doi: 10.3390/jof12010019 (PMC12843489; doi:10.3390/jof12010019)
Supplement: Supplementary file 1 [file jof-12-00019-s001.zip › jof-4030825-supplementary.pdf]

Supplementary Material

Table S1. Studies excluded during the screening process and reasons for exclusion.

No	Author(s) and Year	Study Title	Reason for Exclusion
1	Cabana, H., et al. 2010	Corrigendum to Immobilization of laccase from the white rot fungus Coriolopsis polyzona and use of the immobilized biocatalyst for the continuous elimination of endocrine disrupting chemicals [Bioresource Technology 100 (2009) 3447-3458] (DOI:10.1016/j.biortech.2009.02.052)	Document type: Erratum
2	García-Esquivel, Y., et al. 2023	Effect of particle size, aeration, and substrate height on the production of lignocellulolytic enzymes produced by Trametes polyzona HHM001 grown on corn leaf residues | Efecto del tamaño de partícula, aireación y altura del sustrato sobre la producción de enzimas lignocelulolíticas producidas por Trametes polyzona HHM001 crecido sobre residuos de hojas de maíz	Language: Spanish
3	Abrahão, M.C et al. 2009	Polyporoid (Basidiomycota) in forest fragments of São José do Rio Preto urban perimeter, São Paulo, Brazil | Poliporóides (Basidiomycota) em fragmentos de mata no perímetro urbano de São José do Rio Preto, São Paulo, Brasil	Language: Portuguese
4	Owusu F.W. et al. 2024	Natural durability of Eucalyptus species harvested as electricity transmission poles in two different ecological zones of Ghana	*In press*
5	Sysoeva M.A. et al. 2024	Study of the process of solid-phase cultivation of higher fungi on milled sunflower seeds hulls for the obtaining of composite materials	*In press*
6	Gauthier A. J et al. 2017	Trametes polyzona, an emerging filamentous basidiomycete in Réunion Island	Excluded during title/abstract screening. Medical case report on human infections, not focused on biotechnological applications.
7	Dellière S. et al. 2020	Emerging mould infections: Get prepared to meet unexpected fungi in your patient	Excluded during title/abstract screening. Clinical review listing *T. polyzona* as a potential pathogen, not an applied study.
8	Olusegun O.V. 2014	Molecular identification of trametes species collected from Ondo and Oyo States, Nigeria	Excluded during title/abstract screening. Purely taxonomic and phylogenetic study, not applied research.
9	Nhleko Z.V. et al. 2022	Investigating the impacts of harvest stages, citric acid and calcium lactate treatments on changes in quality attributes and natural microbiota of minimally processed litchi during storage	Excluded during title/abstract screening. *T. polyzona* is part of the natural microbiota on fruit, not the subject of the study.

10	Hafizhasando R. et al. 2021	Fungi in Selo hiking trail of mount Merbabu national park Central Java	Excluded during title/abstract screening. Ecological inventory study, not focused on applications of *T. polyzona*.
11	Amalina D.D. et al. 2023	Enhancing Melon Growth and Nutrient Uptake with Plant-Growth-Promoting Microorganisms from Cucumis melo	Excluded during title/abstract screening. Study on plant growth promotion in agriculture, outside the scope of bioremediation/enzymes.
12	Castillo G. et al. 2004	Correlation between the in vitro growth response to temperature and the habitat of some lignicolous fungi from papua new guinea coastal forests	Excluded during title/abstract screening. Ecological study on fungal growth response, not applied enzymology or biotechnology.
13	Osano A.A. et al. 2004	Biodegradation properties of white rot fungi in Karura Forest, Kenya	Reports not retrieved
14	Kothari R.K. et al. 2005	Decolorization of textile dyes by ligninolytic enzymes of white rot fungus *coriolopsis polysona*	Reports not retrieved
15	Demarche P. et al. 2012	Design-of-experiment strategy for the formulation of laccase biocatalysts and their application to degrade bisphenol A	Reports not retrieved
16	Nerud F. et al. 1991	Ligninolytic properties of different white-rot fungi	Excluded during full-text assessment (Out of topic). General screening study; *T. polyzona* is one of many species without in-depth analysis.
17	Abrahão M.C. et al. 2008	Ligninolytic activity from newly isolated basidiomycete strains and effect of these enzymes on the azo dye orange II decolourisation	Excluded during full-text assessment (Out of topic). Screening study; *T. polyzona* is one of nine strains evaluated, not the focus.
18	Kasonga T.K. et al. 2019	Data on UPLC/MS method validation for the biodegradation of pharmaceuticals and intermediates by a fungal consortium and on T47DK-Bluc reporter gene assay to assess the reduction of their estrogenic activity	Excluded during full-text assessment (Out of topic). Study focuses on analytical method validation and a bioassay for a fungal consortium; *T. polyzona* is only one component without specific analysis of its individual role or properties.
19	Vanhulle S. et al 2007	Cytotoxicity and genotoxicity evolution during decolorization of dyes by White Rot Fungi	Excluded during full-text assessment (Out of topic). *T. polyzona* is one of six strains in a study focused on toxicology of degradation products.
20	Vanhulle S. et al. 2008	Effect of mannan oligosaccharide elicitor and ferulic acid on enhancement of laccases production in liquid cultures of basidiomycetes	Excluded during full-text assessment (Out of topic). *T. polyzona* is a model organism in a study on enzyme induction methodology.

21	Vanhulle S. et al. 2008	Coupling occurs before breakdown during biotransformation of Acid Blue 62 by white rot fungi	Excluded during full-text assessment (Out of topic). *T. polyzona* is one of several strains used to study a specific metabolic pathway for one dye.
22	Jordaan J. et al 2003	Isolation of a thermostable laccase with DMAB and MBTH oxidative coupling activity from a mesophilic white rot fungus	Excluded during full-text assessment (Out of topic). The laccase-producing organism is an unnamed mesophilic fungus, not *T. polyzona.*
23	Arsenault A. et al. 2011	Laccase-based CLEAs: Chitosan as a novel cross-linking agent	Excluded during full-text assessment (Out of topic). *T. polyzona* laccase is a model to test an immobilization technique, not the study's focus.
24	Zimmermann Y.-S. et al. 2011	Sorption-assisted surface conjugation: A way to stabilize laccase enzyme	Excluded during full-text assessment (Out of topic). *T. polyzona* laccase is a model for a novel immobilization method development.
25	Ammann E.M. et al. 2014	Immobilization of defined laccase combinations for enhanced oxidation of phenolic contaminants	Excluded during full-text assessment (Out of topic). *T. polyzona* laccase is one of five combined in a study on immobilization technology.
26	Salas-Veizaga D.M. et al. 2013	Evaluation of the genotoxic potential of reactive black 5 solutions subjected to decolorizing treatments by three fungal strains	Excluded during full-text assessment (Out of topic). *T. polyzona* is one of three strains in a study focused on genotoxicity, not application.
27	Martani F. et al. 2017	The importance of fermentative conditions for the biotechnological production of lignin modifying enzymes from white-rot fungi	Excluded during full-text assessment (Out of topic). A review article on general fermentation conditions, not a primary study on *T. polyzona*.
28	Justo A. Hibbett D.S.2011	Phylogenetic classification of trametes (basidiomycota, polyporales) based on a five-marker dataset	Excluded during full-text assessment (Out of topic). Phylogenetic reclasification study, not focused on applications.
29	Kuswytasari N.D.; et al. 2023	Plastic Biodegradation Potential of Soil Mangrove Mold Isolated from Wonorejo, Indonesia	Excluded during full-text assessment (Out of topic). Screening study; *T. polyzona* is one of eight isolates tested for plastic degradation.
30	Voběrková S. et al. 2018	Immobilization of ligninolytic enzymes from white-rot fungi in cross-linked aggregates	Excluded during full-text assessment (Out of topic). General review article on immobilization techniques; not a primary study on *T. polyzona*.

Table S2. PRISMA 2020 Checklist: Locations of reporting items within the manuscript.

Section and Topic	Item #	Checklist item	Location where item is reported
**TITLE**			
Title	1	Identify the report as a systematic review.	Title Page: The title includes "...and systematic review."
**ABSTRACT**			
Abstract	2	See the PRISMA 2020 for Abstracts checklist.	Abstract: The abstract describes the PRISMA 2020 protocol.
**INTRODUCTION**			
Rationale	3	Describe the rationale for the review in the context of existing knowledge.	Introduction, final paragraph (before "The primary objective..."): Describes the gaps and the need for this review.
Objectives	4	Provide an explicit statement of the objective(s) or question(s) the review addresses.	Introduction, final paragraph: "This review addresses this gap by systematically analyzing the available evidence..."
**METHODS**			
Eligibility criteria	5	Specify the inclusion and exclusion criteria for the review and how studies were grouped for the syntheses.	Section 3.3 ("Overview of systematic literature review (SLR) protocol"): Describes the inclusion/exclusion criteria.
Information sources	6	Specify all databases, registers, websites, organisations, reference lists and other sources searched or consulted to identify studies. Specify the date when each source was last searched or consulted.	Section 3.1 ("Data collection"): "Scopus and Web of Science databases." Appendix A.1, Table A1: Search strategies.
Search strategy	7	Present the full search strategies for all databases, registers and websites, including any filters and limits used.	Appendix A.1, Table A1: Complete search strings for Scopus and WoS. Section 3.1
Selection process	8	Specify the methods used to decide whether a study met the inclusion criteria of the review, including how many reviewers screened each record and each report retrieved, whether they worked independently, and if applicable, details of automation tools used in the process.	Section 3.2: Describes the screening and evaluation process. Figure 1 (PRISMA Flowchart): Illustrates the process.
Data collection	9	Specify the methods used to collect data from	Data extraction was

Section and Topic	Item #	Checklist item	Location where item is reported
process		reports, including how many reviewers collected data from each report, whether they worked independently, any processes for obtaining or confirming data from study investigators, and if applicable, details of automation tools used in the process.	carried out independently by two reviewers using a predefined form. Both reviewers collected the same information and then compared their results. Discrepancies were resolved by discussion. No automation tools were used, and no study authors were contacted.
Data items	10a	List and define all outcomes for which data were sought. Specify whether all results that were compatible with each outcome domain in each study were sought (e.g. for all measures, time points, analyses), and if not, the methods used to decide which results to collect.	The main outcomes extracted were enzymatic activities, biodegradation results, identification of metabolites or bioactive compounds, and reported biological activities. All results relevant to these categories were included as presented in each study. Section 2.2
	10b	List and define all other variables for which data were sought (e.g. participant and intervention characteristics, funding sources). Describe any assumptions made about any missing or unclear information.	Other variables extracted included strain/source, culture or fermentation conditions, substrates, inducers, analytical methods, and any ecotoxicity information. When information was missing or unclear, it was recorded as reported without assumptions. Section 2.2
Study risk of bias assessment	11	Specify the methods used to assess risk of bias in the included studies, including details of the tool(s) used, how many reviewers assessed each study and whether they worked independently, and if applicable, details of automation tools used in the process.	Not applicable (N/A). This review summarizes experimental studies and does not perform risk-of-bias assessment.
Effect measures	12	Specify for each outcome the effect measure(s) (e.g. risk ratio, mean difference) used in the synthesis or presentation of results.	N/A. It is not a review of clinical or intervention trials.
Synthesis methods	13a	Describe the processes used to decide which studies were eligible for each synthesis (e.g. tabulating the study intervention characteristics and comparing against the planned groups for each	Studies were grouped according to the topics defined in the review (general characteristics,

Section and Topic	Item #	Checklist item	Location where item is reported
		synthesis (item #5)).	enzymatic activity, biodegradation, and biological activities). Each study was assigned to the relevant synthesis category based on its reported objectives and results.
	13b	Describe any methods required to prepare the data for presentation or synthesis, such as handling of missing summary statistics, or data conversions.	Data were organized into tables and thematic categories. No data conversions or imputations were needed. Missing information was kept as reported in the original studies.
	13c	Describe any methods used to tabulate or visually display results of individual studies and syntheses.	Results were summarized in tables and figures to display patterns across studies. Tables 2-4
	13d	Describe any methods used to synthesize results and provide a rationale for the choice(s). If meta-analysis was performed, describe the model(s), method(s) to identify the presence and extent of statistical heterogeneity, and software package(s) used.	Section 2.2, 2.3: The synthesis is narrative, organizing the findings of the 61 studies into coherent themes. A narrative synthesis was performed. Findings from the included studies were organized into major themes to summarize trends, methodological differences, and reported outcomes. No meta-analysis was conducted
	13e	Describe any methods used to explore possible causes of heterogeneity among study results (e.g. subgroup analysis, meta-regression).	N/A. No quantitative meta-analysis was performed.
	13f	Describe any sensitivity analyses conducted to assess robustness of the synthesized results.	N/A. Sensitivity analysis does not apply to narrative syntheses.
Reporting bias assessment	14	Describe any methods used to assess risk of bias due to missing results in a synthesis (arising from reporting biases).	N/A. Not applicable to a systematic review of this type.
Certainty assessment	15	Describe any methods used to assess certainty (or confidence) in the body of evidence for an outcome.	N/A. The certainty of the evidence is not assessed.
**RESULTS**			

Section and Topic	Item #	Checklist item	Location where item is reported
Study selection	16a	Describe the results of the search and selection process, from the number of records identified in the search to the number of studies included in the review, ideally using a flow diagram.	Section 2.1 ("Study Selection Results") and Figure 1.
	16b	Cite studies that might appear to meet the inclusion criteria, but which were excluded, and explain why they were excluded.	See **Table S1.** Studies excluded during the screening process and reasons for exclusion.
Study characteristics	17	Cite each included study and present its characteristics.	Section 2.2, 2.3, and References: The included studies are cited and discussed throughout the results section.
Risk of bias in studies	18	Present assessments of risk of bias for each included study.	N/A. Risk of bias was not assessed.
Results of individual studies	19	For all outcomes, present, for each study: (a) summary statistics for each group (where appropriate) and (b) an effect estimate and its precision (e.g. confidence/credible interval), ideally using structured tables or plots.	Individual study results were presented in structured tables summarizing the key findings for each outcome category (enzymatic activity, biodegradation, biological activities, and metabolite identification). Because the included studies used heterogeneous methods and did not provide comparable summary statistics or effect estimates, results were reported as originally presented by each study, without additional statistical calculations.
Results of syntheses	20a	For each synthesis, briefly summarise the characteristics and risk of bias among contributing studies.	Each synthesis section briefly describes the main characteristics of the studies contributing to that topic, including study objectives, experimental conditions, analytical methods, and reported outcomes. As no formal risk-of-bias assessment was performed,

Section and Topic	Item #	Checklist item	Location where item is reported
			methodological limitations and heterogeneity among studies were noted narratively.
	20b	Present results of all statistical syntheses conducted. If meta-analysis was done, present for each the summary estimate and its precision (e.g. confidence/credible interval) and measures of statistical heterogeneity. If comparing groups, describe the direction of the effect.	N/A. There was no statistical synthesis.
	20c	Present results of all investigations of possible causes of heterogeneity among study results.	N/A.
	20d	Present results of all sensitivity analyses conducted to assess the robustness of the synthesized results.	N/A.
Reporting biases	21	Present assessments of risk of bias due to missing results (arising from reporting biases) for each synthesis assessed.	N/A.
Certainty of evidence	22	Present assessments of certainty (or confidence) in the body of evidence for each outcome assessed.	N/A.
**DISCUSSION**			
Discussion	23a	Provide a general interpretation of the results in the context of other evidence.	The findings were interpreted in relation to existing evidence on *Trametes* species and other white-rot fungi. Overall, the results support the relevance of *T. polyzona* for enzymatic production, biodegradation, and applied mycology, showing consistent patterns with previous research in environmental fungal biotechnology.
	23b	Discuss any limitations of the evidence included in the review.	The evidence included in the review showed substantial heterogeneity in experimental conditions, strain identification, analytical methods, and reporting quality. Few studies characterized intermediate metabolites or evaluated ecotoxicity, and methodological inconsistencies limited

Section and Topic	Item #	Checklist item	Location where item is reported
			direct comparison of results.
	23c	Discuss any limitations of the review processes used.	The review relied solely on published studies indexed in selected databases, which may exclude relevant gray literature. No contact with study authors was made to clarify missing information, and a formal risk-of-bias assessment was not conducted due to the experimental nature of the included studies.
	23d	Discuss implications of the results for practice, policy, and future research.	The results highlight the potential of *T. polyzona* for environmental and industrial applications and underscore the need for more standardized methodologies in future research. Improved reporting, consistent analytical approaches, and evaluation of ecotoxicity and metabolite pathways would strengthen the applicability of fungal bioprocesses and support their translation to practice and scale-up.
**OTHER INFORMATION**			
Registration and protocol	24a	Provide registration information for the review, including register name and registration number, or state that the review was not registered.	Section 3.3: "This review was not registered."
	24b	Indicate where the review protocol can be accessed, or state that a protocol was not prepared.	A protocol was not prepared. (Section 3.3)
	24c	Describe and explain any amendments to information provided at registration or in the protocol.	N/A.
Support	25	Describe sources of financial or non-financial support for the review, and the role of the funders or sponsors in the review.	Section "Funding": "Universidad Regional Amazónica Ikiam/ DBM-023-2024".
Competing interests	26	Declare any competing interests of review authors.	Section "Conflicts of Interest": "The authors

Section and Topic	Item #	Checklist item	Location where item is reported
			declare no conflicts of interest."
Availability of data, code and other materials	27	Report which of the following are publicly available and where they can be found: template data collection forms; data extracted from included studies; data used for all analyses; analytic code; any other materials used in the review.	Section "Data Availability Statement": "The data presented in this study is available on request from the corresponding author."

From: Page MJ, McKenzie JE, Bossuyt PM, Boutron I, Hoffmann TC, Mulrow CD, et al. The PRISMA 2020 statement: an updated guideline for reporting systematic reviews. BMJ 2021;372: n71. doi: 10.1136/bmj.n71. This work is licensed under CC BY 4.0. To view a copy of this license, visit https://creativecommons.org/licenses/by/4.0/

Supplementary Table S3.

Table S3. Summary of the main findings on the biological activity of *T.polyzona* extracts reported in the reviewed studies**.**

Biological activity	Main Results *	Refs.
Antioxidant capacity	TPC (mg GAE/ g of extract) = 11.09 - 35.08<br>TEAC (mg Trolox/g of extract) = 0.27 - 3.23<br>DPPH (% inhibition) = 29.59-81.75%	[11]
	ABTS (mg/mL) = 1.89<br>DPPH (mg/mL) = 11.81	[12]
	DPPH, % (10µg/mL) = 17.4. (50µg/mL) = 26.8. (100µg/mL) = 47.9 (500µg/mL) = 72.1. (1000µg/mL) = 75.2	[31]
Antibacterial	Dichlormethane Extract yield (%) = 5.2. IZ (mm). *S. aureus* = 10, *B. subtilis* = 12, *E. Coli* = 10, *P. aeruginosa* = 10, *M. flavus* = 10. / Methanol. Extract yield (%) = 17.8. IZ (mm). *S. Aureus* = 25, *B. subtilis* = 15, *E. Coli* = 20, *P. aeruginosa* = 10, *M. flavus* = 15. / Water. Extract yield (%) = 20.2. IZ (mm). *S. aureus* = 10, *B. subtilis* = 10, *E. coli* = 10, *P. aeruginosa* = 0, *M. flavus* = 10	[31]
Antibacterial	IZ (mm). *K. pneumoniae* ATCC 1100975 = 20.94, ATCC 1002565 = 9.03, BAA1705 = 15.08, ATCC 1100770 = 9.08. *E. coli*. ATCC 700972 = 8.38, ATCC 25922 = 17.22, ATCC 25927 = 16.91. *S. enterica* = 19.34, *S. aureus* = 21.65	[11]
Antibacterial	Ethanolic extract. MIC (SSF/SMF) (mg/mL) = 100, MIC (UFM) (mg/mL) = 200. Highest antistaphylococcal activity in SmF: 28.00 mm. Acetonic extract. MIC (SSF) (mg/mL) = 200, MIC (SmF/UFM) (mg/mL) = 400. GC-MS analysis of the sample confirmed the presence of fourteen bioactive compounds of the fatty acid group	[9]
Anticancer	IC_{50} (mg/mL) = 0.58. Polysacharides of *T. polyzona* suppressed the proliferation of MCF-7 cells, which exhibited >20% inhibition compared with untreated cells (it had cytotoxic activity against the viability of MCF-7 cells)	[12]
Antifungal	IZ (mm). *C. maltosa* = 10. *C. albicans* = 0, *C. krusei* = 0. *A. fumigatus* = 10. *M. sp* = 10. *M. gypseum* = 10. *T. mentagrophytes* = 18	[31]
Anti-inflammatory	Edema (mg) = 7.25. Inhibition (%) = 39.58. The anti-inflammatory effect measured by inhibition of mice ear edema was higher and significantly different ($p \leq 0.05$) than the control	[10]

Lipid peroxidation	IC_{50} (μg/mL) = 222.81. Inhibition of the formation of TBARS by extracts was concentration dependent (90.72% at 562.34 μg/ mL)	[10]
Toxicity	Tolerance to the three ethanolic extracts by *Artemia salina* (concentrations: 10-1000 μg/mL), not exhibited toxicity (n = 30, Lethality = 0 %)	[10]

Notes. * Main Results. IC50: Half-maximal inhibitory concentration. GC-MS: Gas Chromatography Mass Spectrophotometry.

Table S4. Enzymatic activity data by enzyme for each evaluated *T. polyzona* strain

Fungus, Strain	Enzyme activity [c]	Refs.
C. polyzona 38443	CMC = 9 ± 0.8 U/mL<br>Xyl = 7 ± 0.6 U/mL<br>FPA = 1.0 ± 0.1 U/mL<br>Lac = 0.119 ± 0.015 U/mL<br>MnP = 0.454 ± 0.037 U/mL	[32]
C. polyzona 38443	CMC = 5 ± 0.4 U/mL<br>Xyl = 3 ± 0.3 U/mL<br>FPA = 1.2 ± 0.1 U/mL<br>Lac = 0.027 ± 0.002 U/mL<br>MnP = 0.031 ± 0.004 U/mL	[32]
C. polyzona 38443	CMC = 16 ± 2.1 U/mL<br>Xyl = 17 ± 2.0 U/mL<br>FPA = 1.8 ± 0.2 U/mL<br>Lac = 0.173 ± 0.019 U/mL<br>MnP = 0.308 ± 0.022 U/mL	[32]
C. polyzona 38443	CMC = 21 ± 2.1 U/mL<br>Xyl = 23 ± 2.9 U/mL<br>FPA = 3.0 ± 0.3 U/mL<br>Lac = 0.290 ± 0.031 U/mL<br>MnP = 0.375 ± 0.044 U/mL	[32]
C. polyzona 38443	CMC = 32 ± 3 U/mL<br>Xyl = 33 ± 4 U/mL<br>FPA = 2.8 ± 0.3 U/mL<br>Lac = 1.780 ± 0.19 U/mL<br>MnP = 0.896 ± 0.097 U/mL	[32]
C. polyzona 38443	CMC = 94 ± 9 U/mL<br>Xyl = 98 ± 8 U/mL<br>FPA = 5.6 ± 0.6 U/mL<br>Lac = 0.769 ± 0.072 U/mL	[32]

	MnP = 0.516 ± 0.061 U/mL	
C. polyzona 38443	CMC = 14 ± 2 U/mL<br>Xyl = 18 ± 2 U/mL<br>FPA = 1.5 ± 0.2 U/mL<br>Lac = 0.067 ± 0.007 U/mL<br>MnP = 0.046 ± 0.005 U/mL	[32]
C. polyzona 38443	CMC = 39 ± 4 U/mL<br>Xyl = 14 ± 1 U/mL<br>FPA = 1.9 ± 0.2 U/mL<br>Lac = 0.119 ± 0.013 U/mL<br>MnP = 0.124 ± 0.011 U/mL	[32]
T. polyzona BKW-001	EG = 6.89 ± 1.07 U/mL<br>β-Glu = 10.66 ± 5.92 U/mL<br>Exg = 5.91 ± 0.53 U/mL<br>Xyl = 14.17 ± 1.21 U/mL<br>Amy = 56.22 ± 8.58 U/mL<br>Cel = 26.31 ± 2.49 FPU/mL	[17]
T. polyzona BKW-001	EG = 1.97 ± 0.04 U/mL<br>β-Glu = 1 U/mL<br>Exg = 0.2 U/mL<br>Xyl = 0.87 ± 0.05 U/mL<br>Amy = 1,16 ± 0,27 U/mL	[33]
T. polyzona BKW-001	EG = 0.7 ± 0.3 U/mL<br>β-Glu = 0.6 ± 0.2 U/mL<br>Exg = 0.1 ± 0.02 U/mL<br>Xyl = 0.2 ± 0.05 U/mL<br>Amy = 0.5 ± 0.1 U/mL	
T. polyzona BKW-001	EG = 0.3 ± 0.1 U/mL<br>β-Glu = 0.2 ± 0.05 U/mL<br>Exg = 0.05 ± 0.02 U/mL<br>Xyl = 0.3 ± 0.1 U/mL	[33]

	Amy = 0.9 ± 0.2 U/mL	
T. polyzona BKW-001	EG = 0.25 ± 0.1 U/mL<br>β-Glu = 0.15 ± 0.05 U/mL<br>Exg = 0.03 ± 0.01 U/mL<br>Xyl = 0.2 ± 0.05 U/mL<br>Amy = 0.4 ± 0.1 U/mL	[33]
T. polyzona HHM001	Lac = 80 U/mL<br>LiP = 10 U/mL<br>MnP = 18 U/mL.<br>Cel = 3.8 U/mL<br>Xyl = 5 U/mL	[16]
C. polyzona CCBAS 740	MnP = 22 U/mL<br>Lac = 3 U/mL	[34]
T. polyzona WRF03	Lac = 1637 U/mg. MM = 66 kDa	[35]
T. polyzona KU-RNW027	MnP = 16.53 U/mg, MM = 42-44 kDa<br>Lac = 19.72 U/mg. MM = 71 kDa	[14]
T. polyzona WR710–1	Lac = 1.36 U/mg. MM = 71 kDa (SDS-PAGE) / 68 kDa (Gel filtration)	[36]
T. polyzona	Cel = 0.00091 U/mg<br>Xyl = 0.0044 U/mg<br>Cel = 0.001 U/mg<br>Xyl = 0.0036 U/mg	[15]
T. polyzona MPS1-3	Lac = 156.3 U/mL. MM = 31 kDa	[37]
T. polyzona MUCL 38443	Lac = 0.97 U/mL. MM = ~60 kDa	[38]
T. polyzona	Lac = 2.42 U/mL	[39]
T. polyzona RYNF13	MnP = 24 U/mL<br>Lac = 12U/mL<br>LiP = 4 U/mL	[18]
C. polyzona CCBAS 740	MnP = 0.056 U/mL<br>MnIP = 0.023 U/mL<br>Lac = 0.022 Um/L	[40]

	LiP = 0.013 U/mL	
C. polyzona MUCL 38443	Lac = 800 U/L. MM = 54 kDa	[41]
C. polyzona MUCL38443	LiP = 0.022 U/mL<br>MnP = 0.029 U/mL<br>Lac = 0.038 U/mL	[42]
T. polyzona	Lac = 0.0028 U/mL<br>LiP = 0.038 U/mL<br>MnP = 0.019 U/mL	[22]
co-culture of T. polyzona, A. niger, T. longibrachiatum, M. circinelloides and R. microspores	Lac = 0.112 ± 0.010.00 U/mL<br>LiP = 0.095 ± 0.08 U/mL<br>MnP = 0.253.00 ± 0.014 U/mL	[25]

Notes. [c] Enzyme activity. CMC: Carboxymethyl cellulase, Xyl: Xylanase, FPA: Filter paper activity, Lac: Laccase, MnP: Manganese Peroxidase, MnIP: Manganese Independent Peroxidase, LiP: Lignin Peroxidase, Cel: Cellulase, EG: Endoglucanase, Exg: Exoglucanase, Amy: Amylase, β-Glu: Betaglucosidase. MM: Molecular mass.
